# Pathway-Specific Effects of Oral Corticosteroids on Eosinophilic Inflammation and Tissue Remodeling in Chronic Rhinosinusitis with Nasal Polyps

**DOI:** 10.3390/ijms27104565

**Published:** 2026-05-19

**Authors:** Kamil Radajewski, Paweł Burduk, Małgorzata Wierzchowska, Paulina Antosik, Jakub Jóźwicki, Jakub Burduk, Dariusz Grzanka

**Affiliations:** 1 Department of Otolaryngology, Laryngological Oncology and Maxillofacial Surgery, University Hospital No. 2, 85-168 Bydgoszcz, Poland; 2 Department of Otolaryngology, Phoniatrics and Audiology, Collegium Medicum, Nicolaus Copernicus University, 85-168 Bydgoszcz, Poland; 3 Department of Clinical Pathomorphology, Collegium Medicum, Nicolaus Copernicus University, 85-009 Bydgoszcz, Poland

**Keywords:** chronic rhinosinusitis with nasal polyps, oral corticosteroids, eosinophilic inflammation, tissue remodeling, periostin, eotaxin, transforming growth factor-β immunohistochemistry, molecular pathways, precision medicine

## Abstract

Chronic rhinosinusitis with nasal polyps (CRSwNP) is a multifactorial inflammatory disease characterized by heterogeneous phenotypes and endotypes, necessitating personalized therapeutic strategies. Precision medicine approaches integrating molecular biomarkers may improve treatment selection and disease stratification. In this prospective controlled study, we investigated the tissue-level immunohistochemical effects of oral corticosteroids (OCSs) and topical steroids on the expression of periostin, eotaxin, interleukin-4 (IL-4), transforming growth factor-β (TGF-β), and tumor necrosis factor-α (TNF-α) in nasal polyp tissue. Sixty-five patients eligible for endoscopic sinus surgery (ESS) were enrolled and divided into two groups: Group 1 (n = 42) received topical steroids combined with oral prednisone (40 mg/day for 7 days preoperatively), whereas Group 2 (n = 23) received topical steroids alone. Immunohistochemical analysis demonstrated a significant reduction in periostin and eotaxin expression in both epithelial and stromal compartments following OCS therapy, accompanied by increased TGF-β expression. No significant differences were observed in IL-4 or TNF-α expression. These findings indicate that short-term OCSs selectively modulate molecular pathways associated with eosinophilic inflammation and tissue remodeling in CRSwNP, supporting biomarker-driven precision medicine strategies.

## 1. Introduction

Chronic rhinosinusitis with nasal polyps (CRSwNP) is a heterogeneous inflammatory disease characterized by distinct phenotypes and endotypes, reflecting diverse underlying immunological mechanisms. According to EPOS 2020, CRSwNP is defined by the presence of bilateral nasal polyps, symptoms lasting more than 12 weeks, and characteristic findings on nasal endoscopy and computed tomography (CT) imaging [1]. The disease is strongly associated with type 2 inflammation, eosinophilia, epithelial barrier dysfunction, and tissue remodeling, which together contribute to its chronic and recurrent nature [2,3,4].

CRSwNP frequently coexists with asthma, aspirin-exacerbated respiratory disease (AERD), allergic rhinitis, and other systemic inflammatory disorders. These comorbidities significantly influence disease severity, recurrence risk, and treatment response. In recent years, increasing attention has been directed toward precision medicine approaches, in which biomarkers such as periostin, eotaxin, IL-4, IL-5, IL-13, and TGF-β help characterize inflammatory pathways and guide individualized therapy [5,6,7,8,9,10,11,12,13]. It is important to note that CRSwNP endotypes vary geographically: while European, North American, and Australian populations predominantly exhibit Th2-driven inflammation, many East and Southeast Asian cohorts demonstrate Th1- or Th17-skewed immune profiles. Therefore, the present findings apply specifically to a Central European, Th2-predominant population [1,5]. In addition to cytokine profiling, characterization of inflammatory infiltrates using markers such as CD3 (T lymphocytes) and CD68 (macrophages) provides important contextual information about the cellular sources of cytokines and the local immune microenvironment in CRSwNP.

Oral corticosteroids (OCSs) remain an important therapeutic option for reducing inflammation, improving nasal patency, and optimizing surgical conditions in patients with CRSwNP. Systemic corticosteroids modulate Th2-driven inflammation, eosinophil activity, and epithelial remodeling [10,11,12,13], yet their direct impact on the expression of inflammatory mediators within nasal polyp tissue remains insufficiently understood. Our previous study demonstrated that OCSs significantly influence tissue remodeling markers in CRSwNP [14]. However, the effect of OCSs on local cytokine expression—including periostin, eotaxin, IL-4, TGF-β, and TNF-α—has not been fully elucidated [8,9,10,11,12,15,16,17].

### Novelty Statement

This study provides tissue-level immunohistochemical evidence that short-term oral corticosteroids selectively modulate molecular pathways associated with eosinophilic inflammation and tissue remodeling in CRSwNP. We demonstrate coordinated downregulation of periostin and eotaxin alongside increased TGF-β signaling, indicating a differential steroid response between inflammatory and remodeling mediators. These findings refine the molecular understanding of steroid-responsive pathways in CRSwNP and support biomarker-driven precision medicine strategies.

## 2. Results

Our study analyzed 65 patients with CRSwNP. Of these, 42 patients were treated with oral and topical steroids, while 23 patients were treated only with topical steroids. There was no significant difference in age between the two groups, with group 1 having an average age of 47.6 years and group 2 having an average age of 47.1 years. We found a difference in eotaxin expression in epithelial cells and stroma between the two groups. Group 1 had an eotaxin expression of 522.95 ± 621.95 in epithelial cells and 291.69 ± 296.18 in stroma, while group 2 had an expression of 1114.30 ± 751.01 in epithelial cells and 588.91 ± 524.79 in stroma. Additionally, we observed that periostin and TNF-α+/CD68+ were decreased in group 1 (1413.26 ± 507.39/51.43 ± 59.56) compared to group 2 (1623.04 ± 644.27/60.75 ± 63.50). On the other hand, an increase in TGF-β (epithelial cells/stroma) and IL-4 expression was shown in group 1 (1528.15 ± 437.77/827.42 ± 487.81 and 978.99 ± 784.42) compared to group 2 (1232.95 ± 487.02/580.39 ± 451.33 and 850.42 ± 606.22) (Table 1; Table 2).

**Figure 1:**
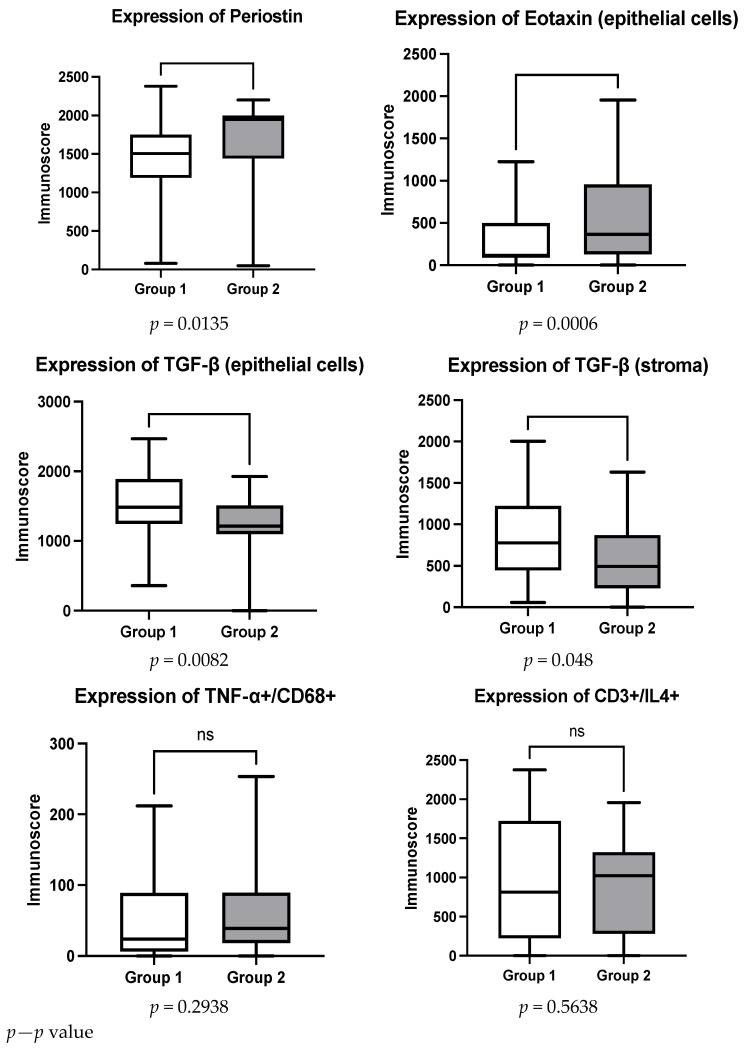
Expression levels of periostin, eotaxin, IL-4, TGF-β, and TNF-α in nasal polyp tissue before and after oral corticosteroid therapy. Data are presented as median with interquartile range. Statistical significance was assessed using the Mann–Whitney U test. ns = not significant; (*p* < 0.05).

We conducted a statistical analysis, the Mann–Whitney test, on the presented results. The test showed that there was a statistically significant decrease in the expression of eotaxin in both epithelial cells and stroma (*p* = 0.0006 and *p* = 0.0116, respectively); a decrease in the expression of periostin (*p* = 0.0135); and an increase in the expression of TGF-β in epithelial cells and stroma (*p* = 0.0082 and *p* = 0.048, respectively). However, no significant differences were found in TNF-α (*p* = 0.2938) and IL-4 (*p* = 0.5638) (Figure 1). These findings indicate that OCSs modulate both epithelial and stromal inflammatory pathways, particularly those associated with eosinophilic inflammation and remodeling.

A post hoc power analysis was performed for the primary outcome (eotaxin expression in epithelial cells). Based on the observed effect size (Cohen’s d = 0.88), sample sizes of 42 and 23 participants, and α = 0.05, the achieved statistical power was 0.83. This indicates that the study was sufficiently powered to detect large between-group differences.

## 3. Discussion

Chronic rhinosinusitis with nasal polyps (CRSwNP) is a heterogeneous inflammatory disease driven by complex interactions between epithelial cells, immune infiltrates, and stromal remodeling processes [8,9]. Although systemic corticosteroids remain a cornerstone of treatment, their tissue-level effects on specific inflammatory and remodeling mediators are incompletely understood. In this prospective controlled study, we demonstrate that short-term oral corticosteroid (OCS) therapy selectively modulates key eosinophil-associated and remodeling-related pathways in nasal polyp tissue.

### 3.1. Steroid-Responsive Type 2 Inflammatory Pathways

Our results show a significant reduction in eotaxin expression in both epithelial and stromal compartments following OCS therapy. Eotaxins are central chemokines responsible for eosinophil recruitment and persistence in CRSwNP and are strongly induced by Th2 cytokines such as IL-4 and IL-13 [18,19,20]. The observed decrease in eotaxin is consistent with the known ability of corticosteroids to suppress eosinophil trafficking and chemotactic signaling, thereby attenuating local type 2 inflammation [10,11,12].

Similarly, periostin expression was significantly reduced after OCS treatment. Periostin is an IL-4/IL-13-induced matricellular protein that promotes eosinophil infiltration, fibrosis, and extracellular matrix (ECM) organization in CRSwNP [14,15]. Previous studies have demonstrated reductions in serum periostin following various anti-inflammatory therapies, including biologics targeting IgE and IL-5 pathways [16,17]. However, data on tissue-level periostin modulation by systemic corticosteroids have been lacking. Our findings provide direct immunohistochemical evidence that OCSs suppress periostin expression within nasal polyp tissue, supporting its role as a steroid-responsive biomarker of eosinophilic inflammation.

### 3.2. Lack of Significant Changes in IL-4 and TNF-α

In contrast to eotaxin and periostin, no significant changes were observed in tissue IL-4 or TNF-α expression. IL-4 is a key upstream Th2 cytokine involved in IgE class switching, eosinophil recruitment, and epithelial activation [18,19]. The absence of a significant tissue-level reduction may reflect differences in cytokine kinetics, compartmentalization, or timing of corticosteroid exposure. While systemic corticosteroids rapidly suppress circulating cytokines, their effects on tissue-resident cytokine expression may be delayed or incomplete.

Similarly, TNF-α, which contributes to early inflammatory signaling and synergizes with IL-4 to enhance eotaxin and VCAM-1 expression [15,16,17], did not show significant modulation. This finding suggests that OCSs preferentially target downstream eosinophil-associated pathways rather than broadly suppressing all pro-inflammatory cytokines at the tissue level.

The inclusion of CD3 and CD68 staining in our immunohistochemical panel provided additional context regarding the inflammatory cell populations present in nasal polyp tissue. CD3-positive T lymphocytes and CD68-positive macrophages represent key cellular sources of cytokines such as IL-4 and TNF-α. Although these markers were not the primary focus of the study, they support the interpretation of cytokine-producing cell subsets and help characterize the broader immune microenvironment in CRSwNP.

### 3.3. Why Did TGF-β Increase? Possible Mechanistic Explanations

A key and unexpected finding of this study was the increased expression of TGF-β in both epithelial and stromal compartments following OCS therapy. TGF-β is a central regulator of tissue repair, fibroblast activation, and ECM remodeling in CRS [20,21,22]. Corticosteroids suppress acute inflammatory gene transcription while simultaneously promoting pathways involved in epithelial stabilization and wound healing, processes in which TGF-β plays a pivotal role.

Importantly, CRSwNP is characterized by reduced TGF-β signaling compared with non-eosinophilic CRS, which has been implicated in impaired collagen deposition and abnormal ECM architecture [23]. Short-term OCSs may partially restore this pathway, shifting the tissue environment toward a remodeling-oriented phenotype. Additionally, eosinophils are a known source of TGF-β, and the higher baseline tissue eosinophilia observed in the OCS group may have contributed to the increased expression [23].

Glucocorticoids act through the glucocorticoid receptor (GR), which translocates to the nucleus and modulates gene transcription by suppressing pro-inflammatory pathways such as NF-κB and AP-1, while simultaneously inducing genes involved in epithelial repair and extracellular matrix organization [10,12,24]. In CRSwNP, where TGF-β signaling is typically reduced compared with non-eosinophilic CRS [23], short-term OCSs may partially restore this pathway, promoting fibroblast activation, collagen deposition, and stabilization of the basement membrane. This shift from an edematous, fibrin-rich phenotype toward a more structurally organized tissue state may explain the observed increase in TGF-β expression.

Taken together, the rise in TGF-β likely represents a steroid-responsive remodeling signature rather than a pro-inflammatory effect, aligning with the clinically observed reduction in edema and improved surgical conditions following preoperative OCSs [25,26].

### 3.4. Implications for Precision Medicine in CRSwNP

The selective modulation of inflammatory mediators observed in this study supports the concept of CRSwNP as a disease composed of multiple molecular endotypes rather than a uniform inflammatory entity [5,6,7]. The reduction in eotaxin and periostin highlights suppression of eosinophil-driven type 2 inflammation, whereas the persistence of IL-4 and TNF-α expression indicates that not all cytokine pathways are equally steroid-responsive at the tissue level [27,28,29]. It should also be emphasized that CRSwNP endotypes differ across global populations, with Th2-dominant inflammation being characteristic of European cohorts such as ours, whereas Asian populations more frequently exhibit Th1- or Th17-skewed profiles [1,5].

From a precision-medicine perspective, identifying which mediators decrease—and which remain unchanged—after OCS therapy may help distinguish steroid-responsive from steroid-resistant inflammatory profiles. Such differentiation is clinically relevant when selecting patients for biologic therapies targeting IL-4/13, IL-5, or IgE pathways. Moreover, the modulation of TGF-β suggests that corticosteroids influence not only inflammation but also structural remodeling, which may have implications for postoperative healing and recurrence risk [21,30]. These findings should be interpreted within the context of a Th2-predominant Central European population and the methodological focus on tissue-level immunohistochemistry without clinical correlation.

## 4. Materials and Methods

**Figure 2:**
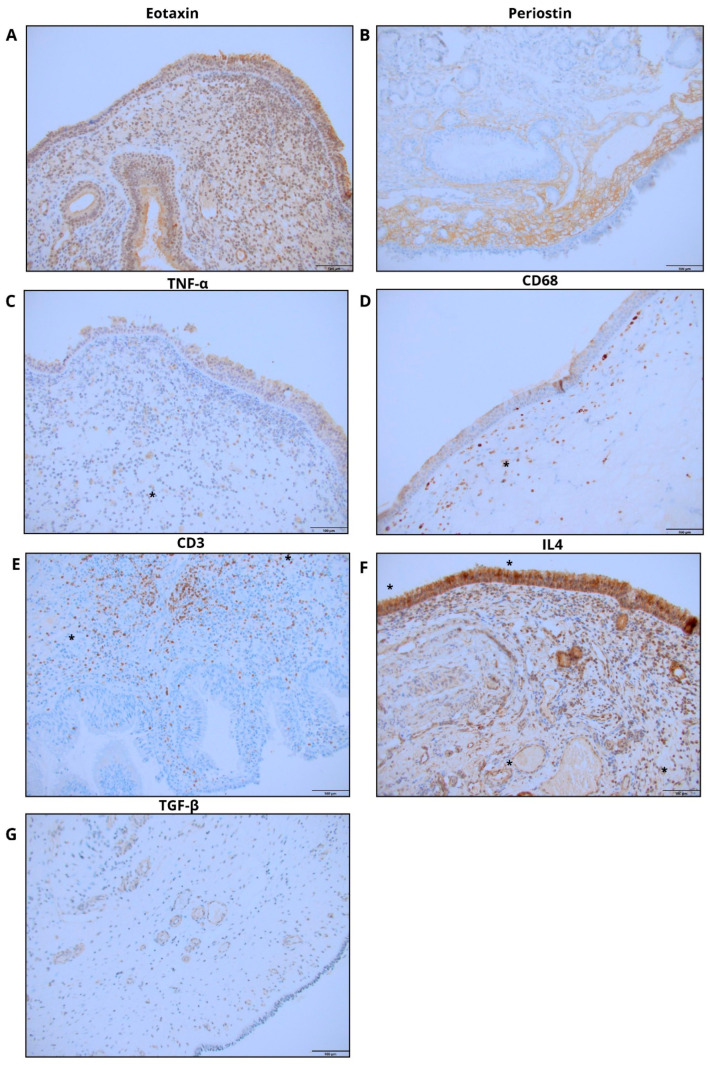
Representative images of immunohistochemical staining for inflammatory and immune markers in nasal polyp tissue. (**A**) Eotaxin expression; (**B**) Periostin expression; (**C**) TNF-α expression; (**D**) CD68-positive macrophages; (**E**) CD3-positive T lymphocytes; (**F**) IL-4 expression; (**G**) TGF-β expression. Primary magnification ×20. The scale bar is 100 µm.

The purpose of this study was to explore how oral steroids (OCSs) and topical steroids affect the expression of periostin, eotaxin, IL-4, TGF-β, and TNF-α in nasal polyp tissue. The study was a prospective controlled study that included patients who had been diagnosed with CRSwNP and had been deemed eligible for endoscopic sinus surgery (ESS)—including uncinectomy, middle meatal antrostomy, anterior and posterior ethmoidectomy, and sphenoidotomy or frontal recess surgery when indicated. The patients had not undergone surgery before. The patients were divided into two groups: Group 1 (n = 42) included patients with CRSwNP who were taking a topical steroid before the procedure (at least four weeks prior), as well as an oral steroid (40 mg per day of prednisone for seven days before the procedure). Group 2 (n = 23) included patients with CRSwNP who were only taking a topical steroid before the procedure (at least four weeks prior). All patients used mometasone furoate nasal spray (200 µg/day), which was the standard topical corticosteroid therapy in our institution during the study period. No patients used corticosteroid drops or budesonide irrigations. After the ESS procedure, the dose of the oral steroid was gradually reduced by 10 mg every five days—only patients in Group 1 received postoperative oral steroid tapering. Patients were enrolled at University Hospital No. 2 in Bydgoszcz, Poland, between January 2017 and December 2020. This study is an extension of our previous study, which assessed the effect of oral corticosteroids on the tissue remodeling of nasal polyps, and focuses on the immunohistochemical diagnosis. The histopathological diagnoses were independently confirmed by two pathologists. The study protocol was approved by the ethics committee of our institution (KB 635/2016 approved on 15 December 2016). This examination was conducted in accordance with the Helsinki Declaration. All patients had previously provided written informed consent for the collection, storage, and future scientific use of tissue samples within the institutional biobank at the time of surgery. The present study included the extended analyses performed on archived biobank material. According to the committee’s decision, no additional written consent was required for this extension, as all procedures were conducted on anonymized, previously collected samples. Formalin-fixed, paraffin-embedded (FFPE) tissue blocks were cut into 3 µm thick sections for immunohistochemical staining, respectively. Immunohistochemical analysis included staining for eotaxin, periostin, TGF-β1, TNF-α, IL-4, as well as CD3 and CD68 to characterize T lymphocyte and macrophage infiltration. For this step, a rotary microtome was used (Accu-Cut^®^ SRM™ 200; Sakura, Torrance, CA, USA). The prepared sections were then transferred onto slides and left for 1 h on a heating plate set at 60 °C. Eotaxin, Periostin, TGF-β1, CD68, TNF-α, CD3, CD4, IL-4 protein expression was detected using the BenchMark^®^ ULTRA automated slide processing system by Roche Diagnostics (Ventana Medical Systems in Tucson, AZ, USA). Tissue sections were incubated with a primary antibody against Eotaxin (diluted 1:300, incubated for 32 min, catalog number: ab133604, Abcam, Cambridge, MA, USA), Periostin (diluted 1:400, incubated for 32 min, catalog number: ab14041, Abcam, Cambridge, MA, USA), TGF-β1 (diluted 1:300, incubated for 32 min, catalog number: ab92486, Abcam, Cambridge, MA, USA), CD68 (ready to use, incubated for 32 min, catalog number: KP-1, Ventana Medical Systems in Tucson, AZ, USA), TNF-α (diluted 1:200, incubated for 32 min, catalog number: ab6671, Abcam, Cambridge, MA, USA), CD3 (ready to use, incubated for 32 min, catalog number: 2GV6, Ventana Medical Systems in Tucson, AZ, USA), CD4 (ready to use, incubated for 32 min, catalog number: SP35, Ventana Medical Systems in Tucson, AZ, USA), IL-4 (diluted 1:900, incubated for 32 min, catalog number: ab9622, Abcam, Cambridge, MA, USA). Antibody staining was visualized using the ultraView Universal DAB Detection Kit from Roche Diagnostics (Ventana Medical Systems in Tucson, AZ, USA). CD3 and CD68 staining were included to quantify the presence of T lymphocytes and macrophages, respectively, and to support interpretation of cytokine-producing cell populations such as TNF-α+/CD68+ and CD3+/IL-4+ cells. Finally, the slides were dehydrated in an alcohol gradient, cleared in xylenes, and mounted with Shandon Consul-Mount (Thermo Scientific, Waltham, MA, USA). Additionally, quality control was performed using positive controls as recommended by the manufacturer and in accordance with the literature. The immunohistochemical evaluation of protein expression was performed in a blinded fashion by two independent pathologists using a light ECLIPSE E400 microscope (Nikon Instruments Europe, Amsterdam, The Netherlands) at 20× and 40× magnification. The scoring system for proteins was determined by adding the multiplication of the fraction of stained cells (FSC) and the percentage of cells at each staining intensity level with the staining intensity ordinal value (scored from 0 for “no staining” to 3+ for “strong staining”), according to a modified H-score with the formula: [1 × (FSC × % cells 1+) + 2 × (FSC × % cells 2+) + 3 × (FSC × % cells 3+)], whereby FSC was calculated based on the number of stained cells per 1000 cells of the same type (range from 0 to 3000) (Figure 2). Statistical analysis was performed using GraphPad Prism version v9.5.1 (GraphPad Software, San Diego, CA, USA). The normality of data distribution was assessed with the Shapiro–Wilk test. Comparative analysis was carried out with the Mann–Whitney test and Kruskal–Wallis test for continuous variables. The correlations between protein expressions were evaluated by utilizing the Spearman correlation coefficient. A significance level of *p* < 0.05 was considered statistically significant.

## 5. Limitations

This study has several limitations. First, no a priori sample size calculation was performed, as the study was based on a consecutive real-world surgical cohort; therefore, the relatively small and uneven group sizes may have limited the ability to detect more subtle differences between treatment groups. Second, the single-center design may reduce the generalizability of the findings.

Detailed baseline characteristics such as disease duration, comorbid asthma or AERD, and standardized preoperative symptom scores (e.g., SNOT-22) were not systematically recorded in a structured format for all patients. This limitation reflects the design of the original ethics-approved protocol, which focused exclusively on histopathological and immunohistochemical evaluation and served as a continuation of our previous study on tissue remodeling (JCM 2021) [31]. As a result, comprehensive baseline profiling and clinical–molecular correlations could not be performed.

CT imaging was performed as part of routine preoperative ESS qualification and not for research purposes; therefore, no additional radiation exposure was introduced by the study. During the recruitment period (2017–2020), CBCT was not routinely used in our institution for CRS assessment.

The study protocol did not include standardized assessment of surgical field visualization or systematic evaluation of symptom improvement after OCS therapy. Consequently, correlations between biomarker changes and clinical outcomes could not be assessed. Future prospective studies should integrate both molecular profiling and standardized clinical outcome measures to enhance translational relevance.

The study did not include untreated controls or patients receiving biologics or antibiotics, which limits the ability to directly compare corticosteroid effects with other therapeutic modalities. Finally, despite standardized laboratory procedures, potential variability in immunohistochemical quantification cannot be fully excluded.

## 6. Conclusions

Short-term oral corticosteroid therapy resulted in a significant decrease in periostin and eotaxin expression and an increase in TGF-β expression in nasal polyp tissue, while TNF-α and IL-4 levels remained unchanged. These findings indicate that OCSs selectively modulate eosinophil-associated inflammatory pathways and remodeling-related mediators rather than uniformly suppressing all cytokine signals. The observed upregulation of TGF-β may reflect steroid-induced activation of epithelial repair and extracellular matrix organization. Overall, the results support the concept of differential steroid responsiveness among molecular pathways in CRSwNP and highlight the potential value of biomarker-based precision medicine. These findings should be interpreted within the context of a Th2-predominant Central European population and the methodological focus on tissue-level immunohistochemistry without clinical correlation.

## Figures and Tables

**Table 1:** Patient characteristic.

Parameter	Group 1	Group 2
Age	47.67 ± 13.31	47.13 ± 11.94
Eotaxin (epithelial cells)	522.95 ± 621.95	1114.30 ± 751.01
Eotaxin (stroma)	291.69 ± 296.18	588.91 ± 524.79
Periostin	1413.26 ± 507.39	1623.04 ± 644.27
TGF-β (epithelial cells)	1528.15 ± 437.77	1232.95 ± 487.02
TGF-β (stroma)	827.42 ± 487.81	580.39 ± 451.33
TNF-α+/CD68+	51.43 ± 59.56	60.75 ± 63.50
CD3+/IL-4+	978.99 ± 784.42	850.42 ± 606.22

TGF-β—Transforming Growth Factor Beta, TNF-α—Tumor Necrosis Factor Alpha, CD68—Cluster of Differentiation 68, CD3—Cluster of Differentiation 3, IL-4—Interleukin 4.

**Table 2:** Summary of immunohistochemical marker expression in CRSwNP patients treated with oral corticosteroids (Group 1) versus topical steroids alone (Group 2).

Marker	Group 1 (OCS + Topical) Mean ± SD	Group 2 (Topical Only) Mean ± SD	*p*-Value	Direction of Change
Eotaxin (epithelium)	522.95 ± 621.95	1114.30 ± 751.01	0.0006	↓
Eotaxin (stroma)	291.69 ± 296.18	588.91 ± 524.79	0.0116	↓
Periostin	1413.26 ± 507.39	1623.04 ± 644.27	0.0135	↓
TGF-β (epithelium)	1528.15 ± 437.77	1232.95 ± 487.02	0.0082	↑
TGF-β (stroma)	827.42 ± 487.81	580.39 ± 451.33	0.0480	↑
TNF-α+/CD68+	51.43 ± 59.56	60.75 ± 63.50	0.2938	=
CD3+/IL-4+	978.99 ± 784.42	850.42 ± 606.22	0.5638	=

↓ decreased in Group 1; ↑ increased in Group 1; = no significant change.

## Data Availability

The data supporting the findings of this study are not publicly available due to privacy and ethical restrictions. No new datasets were generated or analyzed during the current study beyond what is included in the manuscript.

## Abbreviations

The following abbreviations are used in this manuscript:

CRS	Chronic Rhinosinusitis
CRSwNP	Chronic Rhinosinusitis with Nasal Polyps
ESS	Endoscopic Sinus Surgery
OCSs	Oral Corticosteroids
IL-4	Interleukin 4
IL-5	Interleukin 5
IL-13	Interleukin 13
TNF-α	Tumor Necrosis Factor Alpha
TGF-β	Transforming Growth Factor Beta
VCAM-1	Vascular Cell Adhesion Molecule 1
ECM	Extracellular Matrix
IgE	Immunoglobulin E
Th2	T helper type 2 cells
CD3	Cluster of Differentiation 3
CD68	Cluster of Differentiation 68
